# Perioperative management of polycythemia vera with advanced gastric cancer: A case report

**DOI:** 10.1016/j.ijscr.2019.04.022

**Published:** 2019-04-16

**Authors:** Tetsuya Mochizuki, Kazuaki Tanabe, Ryusuke Saito, Hiroshi Ota, Yuji Yamamoto, Yoshihiro Saeki, Hideki Ohdan

**Affiliations:** Department of Gastroenterological and Transplant Surgery, Graduate School of Biochemical & Health Sciences, Hiroshima University, 1-2-3 Kasumi, Minami-ku, Hiroshima, 734-8551, Japan

**Keywords:** CA19-9, carbohydrate antigen 19-9, CEA, carcinoembryonic antigen, Hct, hematocrit, Plt, platelet, PV, polycythemia vera, WBC, white blood cell, Gastric cancer, Polycythemia vera, Gastrectomy

## Abstract

**Introduction:**

Polycythemia vera (PV) is a chronic myeloproliferative disorder with generally good prognosis. However, thrombotic and cardiovascular complications are among the leading causes of death in patients with PV and sufficiently effective management strategies are yet to be established. We report a case involving operation for gastric cancer in a patient being treated for PV.

**Presentation of case:**

A 73-year-old man was diagnosed as PV on the basis of abnormal laboratory data eight years previously. Three months ago, he was referred to our department for anorexia and melena. The preoperative diagnosis was advanced gastric cancer, Stage IIB. To avoid perioperative thrombotic and hemorrhagic events, low-dose aspirin administration was continued with an additional dose of hydroxyurea. Emergent control was necessary because of the severity of gastric cancer symptoms, including pyloric stenosis and anemia. Distal gastrectomy with D2 lymph node resection was performed. The postoperative course was satisfactory, and the patient is currently doing well without any signs of recurrence or hematological complications.

**Discussion:**

We described the successful management of a patient being treated for PV who underwent gastrectomy. As mentioned earlier, thrombotic complications and hemorrhage during the perioperative period are the major risk factors in patients with PV. In this case, control of white blood cell and platelet counts during the perioperative period led to good results.

**Conclusions:**

Perioperative management for PV is important for complication-free surgery. Careful follow up should be performed for gastric cancer and PV recurrence.

## Highlights

• We report a case involving operation for gastric cancer after treatment for PV.
• Control of WBC and Plt counts during the perioperative period led to good results.
• Perioperative management for PV is important for complication-free surgery.
• Careful follow up should be performed for gastric cancer and PV recurrence.

## 1 Introduction

This work has been reported in line with the SCARE criteria [1]. Polycythemia vera (PV) is a chronic myeloproliferative disorder characterized by a clonal excess of erythrocytes, leukocytes, and platelets [2]. The incidence rate of PV is 2.3 per 100,000 person-years, and the 5-year survival with myelosuppressive therapy is approximately 85% [3,4]. Patients with PV experience a chronic clinical course with increasing risk of thrombosis; in some patients, PV progresses to myelofibrosis with myeloid metaplasia or acute leukemia [5]. The prognosis PV is good; however, there is a risk of thrombo-hemorrhagic events. Thrombotic and cardiovascular complications are among the leading causes of death in patients with PV [6].

The incidence of hematological complications is reportedly extremely high during the perioperative period and sufficiently effective management strategies are yet to be established [7,8]. To avoid such perioperative complications, we propose monitoring and controlling three hematological markers—hematocrit level (Hct), white blood cell (WBC) count, and platelet count (Plt). Herein, we present a case involving a patient who underwent gastrectomy with treatment of PV.

## 2 Presentation of case

A 73-year-old man was diagnosed with PV based on abnormal laboratory data eight years previously and was undergoing treatment. At the first visit to the Department of Hematology, his hemoglobin level was 21.6 g/dL, Hct was 62.9%, WBC count was 94,800 /mL, and Plt count was 612,000 /mL. A mutation in the Janus Kinase 2 gene (JAK2V617 F)—identified in at least 95% of patients with PV—was identified in our case. Treatment with hydroxyurea, 500 mg every alternate day, and aspirin, 100 mg every day, was initiated. Although the hemoglobin level and the leukocyte count decreased to 14.4 g/dL and 36,220 /mL, respectively, the Plt count could not be controlled well.

**Fig. 1:**
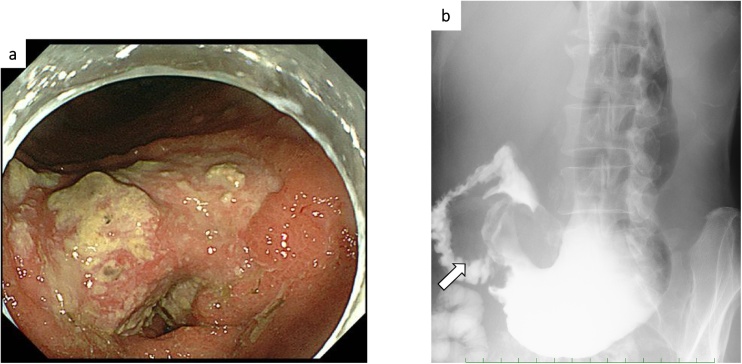
Gastroscopy findings: a. Gastroscopy revealed type 3 tumor in the antrum of the stomach and pyloric stenosis. b. Upper gastrointestinal series showed advanced gastric cancer located in the antrum of the stomach (arrow).

**Fig. 2:**
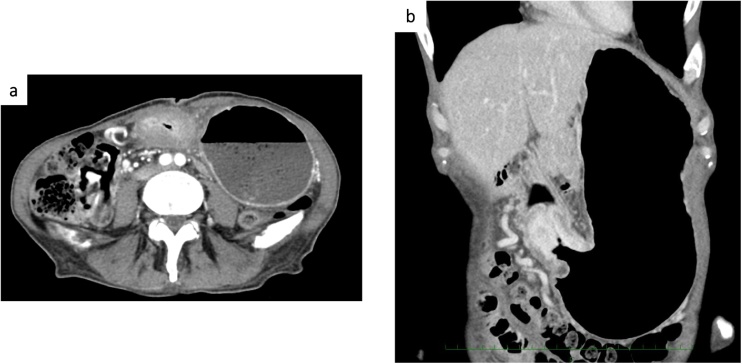
Enhanced abdominal computed tomography findings. Computed tomography revealed enhanced thickening of the stomach wall and pyloric stenosis.

He referred to our department for anorexia and melena three month previously. Laboratory examination showed severe anemia (Hb 6.3 g/dL) and a gastrointestinal endoscopy procedure was performed. Gastroscopy revealed a type 3 tumor in the antrum of the stomach with pyloric stenosis (Fig. 1). Poorly differentiated adenocarcinoma was detected via biopsies from the lesion. Thoraco-abdominal computed tomography revealed enhanced thickening of the stomach wall, but no metastatic lesion or lymph node swelling could be detected (Fig. 2). The levels of tumor markers, including carcinoembryonic antigen (CEA) and carbohydrate antigen 19-9 (CA19-9), were within the normal range. The preoperative diagnosis was advanced gastric cancer, categorized as Stage IIB according to the Japanese Classification of Gastric carcinoma (T4a (SE)N0M0) [9].

To avoid perioperative complications, aspirin intake was continued and the Plt level decreased to normal by additional intake of hydroxyurea. Subsequently, distal gastrectomy with D2 lymph node resection was performed. On laparotomy, tumor invasion into the transverse colon was strongly suspected, so an additional partial colectomy procedure was performed. Cytological results were negative and no metastatic lesion was detected. Tumor invasion into the transverse colon was discounted on the basis of pathological findings and the final stage was categorized as pStage IIIC (T4aN3aM0).

**Fig. 3:**
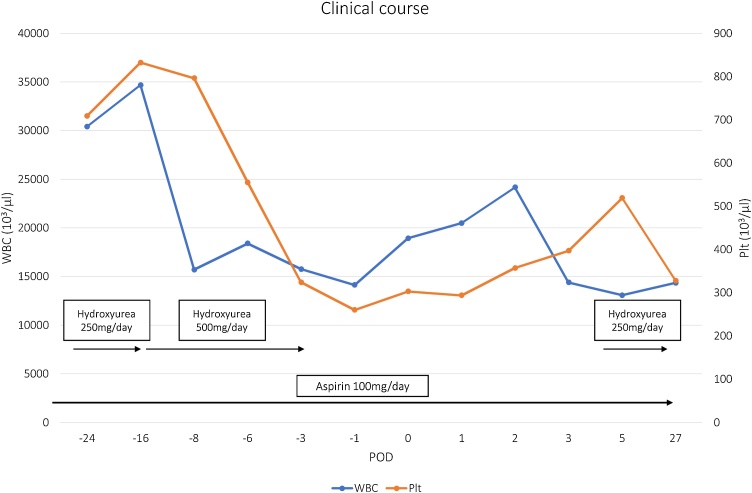
Control of WBC and Plt counts: WBC and Plt counts were well controlled during the perioperative period by taking low-dose-aspirin and hydroxyurea.

Hydroxyurea was re-administered 7 days after surgery and WBC and platelet counts were well controlled during the perioperative period (Fig. 3). The patient was discharged on postoperative day 14 without any complications. S-1 (fluoropyrimidine) was administered for adjuvant chemotherapy [10,11] and there has been no sign of gastric cancer recurrence over 10 months.

The patient was provided a thorough explanation of the publication process and he provided his permission to publish this report.

## 3 Discussion

We have described the successful management of a patient being treated for PV who underwent gastrectomy. As mentioned earlier, thrombotic complications and hemorrhage during the perioperative period are the major risk factors in patients with PV [12]. In this case, control of WBC and Plt counts during the perioperative period led to good results.

In a study involving 255 patients with PV or essential thrombocytopenia, Ruggeri et al. reported that the incidence of venous thromboembolisms was 3.9%, and that of arterial thromboembolisms was 3.8% during the postoperative period. They also reported that the incidence of bleeding complications was 10.5%, and 23 patients (7.3%) experienced major hemorrhage [12]. The hematocrit level (Hct) and WBC count have been reported as risk factors for thrombotic events [6,8,[13], [15]]. Marchioli et al. reported that patients with Hct target <45% had lower rates of thrombotic complications compared with patients with Hct target of 45–50% [8]. Leukocytosis is a potential risk factor for thrombosis. A WBC count >15 × 10^9^ /L has been identified as a significant risk factor for myocardial infarction [13]. Thus, control of these parameters is necessary during the perioperative period.

Given the risk of hemorrhage, control of the Plt count is the most important factor in the management of patients with PV. In the PSVG-05 trial, a higher Plt count tended to be associated with a higher risk of hemorrhage [16]. Hemorrhage in patients with myelodysplastic disorders occurs owing to the decrease of large multimers of the von Willebrand factors when the Plt count is extremely high— a condition called acquired von Willebrand syndrome [17]. Low-dose of aspirin effectively suppresses the Plt count. There was no association between hemorrhage and antithrombotic therapy [12,18].

The standard treatment for patients with PV is anti-platelet therapy and myelosuppressive therapy with hydroxyurea [18,19]. Treatment with hydroxyurea is widely used as the first-line therapy for high-risk patients. Hydroxyurea is a myelosuppressive drug with low toxicity [20]. In >60-year-old patients at high risk of thrombotic events, hydroxyurea plus low-dose aspirin is recommended [19].

Most of the previous cases of surgical treatment for PV patients are elective surgery and they have plenty of time to disease control of PV. However, in our case, operation was performed emergently and there was not enough time to control PV. In addition, it was impossible to evaluate PV condition by Hb or Hct because of anemia, so we used WBC and Plt count intstead. That lead to good result of surgery without any complication.

## 4 Conclusions

In conclusion, our study highlights that for reducing perioperative complications, the most important aspects of the perioperative management of patients with PV include the normalization of Hct, WBC, and Plt counts. In our case, the Hct level was maintained within the normal range owing to bleeding from the tumor; moreover, the WBC and Plt counts were well controlled with an increase of hydroxyurea and low-dose-aspirin. Emergent control was necessary because of pyloric stenosis and bleeding from gastric cancer. Perioperative management for PV is important for complication-free surgery. Careful follow up should be performed for gastric cancer and PV recurrence.

## Conflicts of interest

None of the authors have any commercial or financial involvement in connection with this study that represents or appears to represent any conflicts of interest.

## Sources of funding

This research did not receive any specific grant from funding agencies in the public, commercial, or not-for-profit sectors.

## Ethics approval

We have a consent by the patient. Ethical approval was obtained from the ethical committee of Hiroshima University Hospital.

## Consent

Written informed consent was obtained from the patient for publication of this case report and any accompanying images.

## Author contribution

All authors in this manuscript contributed to the interpretation of data, and drafting and writing of this manuscript. Tetsuya Mochizuki is first author of this paper. Kazuaki Tanabe is corresponding author of this paper. Tetsuya Mochizuki, Ryusuke Saito and Kazuaki Tanabe conceived and designed the study and drafted the manuscript. Tetsuya Mochizuki, Ryusuke Saito, Hiroshi Ota, Yuji Yamamoto, Yoshihiro Saeki, Kazuaki Tanabe, and Hideki Ohdan were engaged in patient’s care in our hospital including surgery. Kazuaki Tanabe contributed to study concept, and review of the final manuscript and submission of the paper. All the authors read and approved the final manuscript.

## Registration of research studies

The manuscript does not report the result of an experimental investigation or research on human subjects.

## Guarantor

Kazuaki Tanabe.

## Provenance and peer review

Not commissioned, externally peer-reviewed.

## References

[1] Agha R.A., Borrelli M.R., Farwana R., Koshy K., Fowler A.J., Orgill D.P. (2018). The SCARE 2018 statement: updating consensus surgical CAse REport (SCARE) guidelines. Int. J. Surg..

[2] Tefferi A. (2012). Polycythemia vera and essential thrombocythemia: 2012 update on diagnosis, risk stratification, and management. Am. J. Hematol..

[3] Fitts W.T., Erde A., Peskin G.W., Frost J.W. (1960). Surgical implications of polycythemia vera. Ann. Surg..

[4] Ania B.J., Suman V.J., Sobell J.L., Codd M.B., Silverstein M.N., Melton L.J. (1994). Trends in the incidence of polycythemia vera among Olmsted County, Minnesota residents, 1935-1989. Am. J. Hematol..

[5] Spivak J.L. (2002). Polycythemia vera: myths, mechanisms, and management. Blood.

[6] Kroll M.H., Michaelis L.C., Verstovsek S. (2015). Mechanisms of thrombogenesis in polycythemia vera. Blood Rev..

[7] Barbui T., Barosi G., Birgegard G., Cervantes F., Finazzi G., Griesshammer M. (2011). Philadelphia-negative classical myeloproliferative neoplasms: critical concepts and management recommendations from European LeukemiaNet. J. Clin. Oncol..

[8] Marchioli R., Finazzi G., Specchia G., Cacciola R., Cavazzina R., Cilloni D. (2013). Cardiovascular events and intensity of treatment in polycythemia vera. N. Engl. J. Med..

[9] Japanese Gastric Cancer Association (2011). Japanese classification of gastric carcinoma: 3rd English edition. Gastric Cancer.

[10] Sakuramoto S., Sasako M., Yamaguchi T., Kinoshita T., Fujii M., Nashimoto A. (2007). Adjuvant chemotherapy for gastric cancer with S-1, an oral fluoropyrimidine. N. Engl. J. Med..

[11] Sasako M., Sakuramoto S., Katai H., Kinoshita T., Furukawa H., Yamaguchi T. (2011). Five-year outcomes of a randomized phase III trial comparing adjuvant chemotherapy with S-1 versus surgery alone in stage II or III gastric cancer. J. Clin. Oncol..

[12] Ruggeri M., Rodeghiero F., Tosetto A., Castaman G., Scognamiglio F., Finazzi G. (2008). Postsurgery outcomes in patients with polycythemia vera and essential thrombocythemia: a retrospective survey. Blood.

[13] Landolfi R., Di Gennaro L., Barbui T., De Stefano V., Finazzi G., Marfisi R. (2007). Leukocytosis as a major thrombotic risk factor in patients with polycythemia vera. Blood.

[15] Schafer A.I. (2006). Molecular basis of the diagnosis and treatment of polycythemia vera and essential thrombocythemia. Blood.

[16] Di Nisio M., Barbui T., Di Gennaro L., Borrelli G., Finazzi G., Landolfi R. (2007). The haematocrit and platelet target in polycythemia vera. Br. J. Haematol..

[17] Budde U., Scharf R.E., Franke P., Hartmann-Budde K., Dent J., Ruggeri Z.M. (1993). Elevated platelet count as a cause of abnormal von Willebrand factor multimer distribution in plasma. Blood.

[18] Landolfi R., Marchioli R., Kutti J., Gisslinger H., Tognoni G., Patrono C. (2004). Efficacy and safety of low-dose aspirin in polycythemia vera. N. Engl. J. Med..

[19] Harrison C.N., Campbell P.J., Buck G., Wheatley K., East C.L., Bareford D. (2005). Hydroxyurea compared with anagrelide in high-risk essential thrombocythemia. N. Engl. J. Med..

[20] Cortelazzo S., Finazzi G., Ruggeri M., Vestri O., Galli M., Rodeghiero F. (1995). Hydroxyurea for patients with essential thrombocythemia and a high risk of thrombosis. N. Engl. J. Med..

